# Semi-coke briquettes: towards reducing emissions of primary PM_2.5_, particulate carbon, and carbon monoxide from household coal combustion in China

**DOI:** 10.1038/srep19306

**Published:** 2016-01-19

**Authors:** Qing Li, Xinghua Li, Jingkun Jiang, Lei Duan, Su Ge, Qi Zhang, Jianguo Deng, Shuxiao Wang, Jiming Hao

**Affiliations:** 1 State Key Joint Laboratory of Environment Simulation and Pollution Control, School of Environment, Tsinghua University, Beijing, 100084, China; 2 School of Chemistry and Environment, Beihang University, Beijing, 100191, China; 3 State Environmental Protection Key Laboratory of Sources and Control of Air Pollution Complex, Beijing, 100084, China; 4 College of Environmental Science and Engineering, Nankai University, Tianjin, 300071, China

## Abstract

Direct household use of unprocessed raw coals for cooking and heating without any air pollution control device has caused serious indoor and outdoor environment problems by emitting particulate matter (PM) and gaseous pollutants. This study examined household emission reduction by switching from unprocessed bituminous and anthracite coals to processed semi-coke briquettes. Two typical stoves were used to test emission characteristics when burning 20 raw coal samples commonly used in residential heating activities and 15 semi-coke briquette samples which were made from bituminous coals by industrial carbonization treatment. The carbonization treatment removes volatile compounds from raw coals which are the major precursors for PM formation and carbon emission. The average emission factors of primary PM_2.5_, elemental carbon, organic carbon, and carbon monoxide for the tested semi-coke briquettes are much lower than those of the tested raw coals. Based on the current coal consumption data in China, switching to semi-coke briquettes can reduce average emission factors of these species by about 92%, 98%, 91%, and 34%, respectively. Additionally, semi-coke briquette has relatively lower price and higher burnout ratio. The replacement of raw coals with semi-coke briquettes is a feasible path to reduce pollution emissions from household activities.

China has been experiencing severe haze pollutions^1^,^2^. Over the past decade, 14 million tons of PM_2.5_ (particulate matter of aerodynamic diameter equal to or less than 2.5 μm)^3^ and 2 million tons of black carbon (BC)^4^,^5^ were annually emitted to the atmosphere from anthropogenic activities in China, approximately one third and 36% of the global anthropogenic emissions, respectively^3^,^4^,^5^. These emissions and pollution they caused have been influencing human health^6^ and global climate, for example, aerosols primarily scatter solar radiation and their BC components absorb solar radiation^7^,^8^. In response to the severe pollution, the Chinese State Council announced its aim to reduce PM_2.5_ concentrations in Beijing-Tianjin-Hebei area, Yangtze River Delta, and Pearl River Delta by 25%, 20%, and 15% from 2012 to 2017, respectively^8^. Together with the US government, China announced that it will peak its carbon emissions by 2030 and will get 20% of its energy from zero-carbon emission sources by that time^9^. The control of air pollution is in race with the economy development in China, and efforts have been made to limit anthropogenic emissions^10^, such as requiring coal-fired power plants to install selective catalytic reduction (~80% were installed by 2014), electrostatic and bag-filtering dust precipitators (~100% by 2012), and flue gas desulphurization systems (~100% by 2012)^11^,^12^,^13^,^14^,^15^.

China is the largest coal producer and consumer (3888 Mt in 2013, ~49.7% in the world, see Fig. 1)^16^. Coal combustion is the largest contributor to China’s PM_2.5_ pollution^17^. Though efforts have been made to reduce emissions from industrial coal consumption, pollutant emission factors from household coal combustion (without any air pollution control devices) can be ~100 times higher than those from power plant coal boilers^18^. Contribution of primary PM_2.5_ emission from household coal combustion is more than 50% of total emission from all coal consumption in China since 2009 (see Fig. 1). The contribution of household coal combustion to China’s total BC emissions is ~27.5% in 2007 (the largest contributor)^4^. Due to the limited storage of petroleum and natural gas and relatively abundant coal storage (with relatively low price) in China, it is predicted that this coal-based energy structure will last for decades, especially in rural areas^14^,^17^. Since 2012, Beijing government has been making new policies to reduce emissions from residential coal consumption, e.g., promoting new stoves and replacing low rank coal (mainly bituminous coal) with anthracite with the help of financial subsidy^19^,^20^. Unfortunately, anthracite is not well accepted due to its difficulty in ignition, low burnout ratio in domestic stove, and high price. Semi-coke coal, an industrial by-product made from low rank coal by low temperature carbonization, is also considered as the household fuel by Beijing, Tianjin, and Hebei governments.

Emission characteristics are important information for policy maker to develop policy on household energy usage. Due to incomplete combustion, household coal consumption generates large amount of particulate matter (PM), particulate carbon including elemental carbon (EC, commonly regarded as equivalent to BC^21^,^22^) and organic carbon (OC), and carbon monoxide (CO). EC and OC are two dominant species in particulate matter emitted from household coal combustion^21^, e.g., the contribution of particulate carbon in primary PM_2.5_ mass when burning bituminous coal is higher than 80%^18^. PM emission factor (EF) is governed by geological maturity of coal, burning form of coal, and burning efficiency of coal stoves^23^,^24^,^25^,^26^,^27^,^28^,^29^,^30^,^31^. Geological maturity (from bituminous coal to anthracite) was reported to a dominant parameter in PM emission^23^,^25^,^32^,^33^. Low rank bituminous coal has higher volatile content than high rank anthracite coal. Volatile compounds are precursors for PM formation during residential coal combustion with low combustion efficiency. Accordingly, primary PM_2.5_ EF of bituminous coal can be dozens of that of anthracite coal^23^,^32^,^34^. Semi-coke coal has low volatile content (similar level to that of anthracite coal) because of the carbonization processing, though its emission characteristics have not been reported.

Previously reported household EFs of primary PM_2.5_, EC, OC, and CO in China vary several orders of magnitude due to testing in various stoves operating under different controlled conditions^21^,^27^,^29^,^30^,^35^,^36^. To evaluate environmental impacts from burning different household coals and to develop household fuel policy in China, systematic evaluation of EFs under the same controlled conditions are needed. After characterizing pollutant emissions from burning raw coals and processed coals in residential heating stoves, we here present a story of replacing currently consumed raw bituminous and anthracite coals with carbonization processed residential coal, i.e., semi-coke briquette. Feasibility analysis is also performed with regarding to burnout ratio, price, and semi-coke production capacity in China.

## Results

### Emission characterization

#### Emission profiles during residential coal combustion

**Figure 2:**
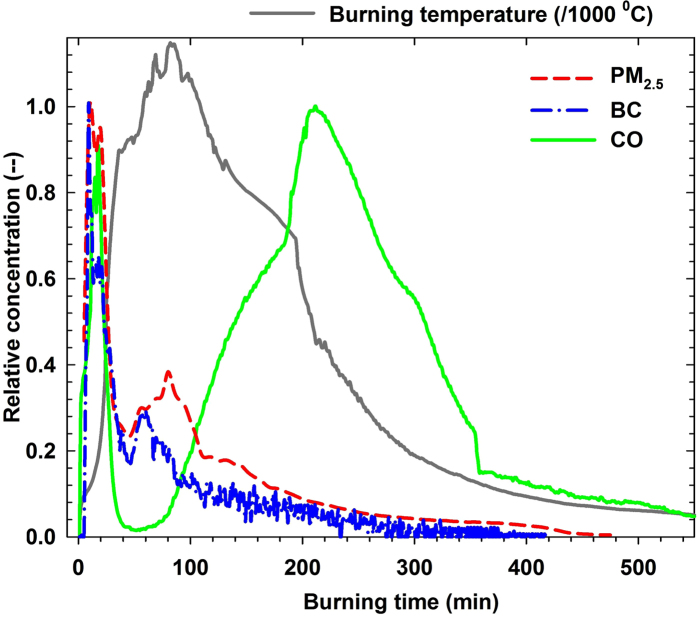
Typical emission profiles of PM_2.5_, BC, and CO with reference to the burning temperature from fire start to fire extinction (sample S9br in Laowan stove). For better comparison, concentrations were normalized by their highest concentrations. Temporal PM_2.5_ concentrations were estimated from measured particle size distributions. Temporal BC concentrations were measured by the BC monitor.

Figure 2 shows typical emission profiles of primary PM_2.5_, BC, and CO with reference to the burning temperature from fire start to fire extinction when burning a semi-coke briquette (S9br) in Laowan stove. Other samples have similar emission profiles (typical profiles for anthracite and bituminous coal combustions are shown in Figs S2 and S3, respectively). Most primary PM_2.5_ and BC were emitted during coal heating and initial-burning period during which most volatile compounds were vaporized from coal samples. The BC profile is consistent with previous report^35^. This suggests that most particulate matter and its carbon component are possibly originated from the incomplete combustion of volatile compounds in coals. Owing to higher content of volatile compounds in bituminous coals than those in anthracite and semi-coke coals, bituminous coals have much higher EFs of primary PM_2.5_, EC, and OC (see Tables S3 and S4) and higher primary PM_2.5_ mass concentration in the flue gas (see Fig. S4). There was a sharp peak of CO at the ignition stage and a broad emission peak at the char burning stage. Both peaks occurred at relatively low burning temperature. Two CO peaks were also reported during smog chamber study of the residential coal combustion and explained as the incomplete combustion of volatile matter and inertinite^37^. The peak at the char burning stage was also contributed by the barrier of generated ashes which cover unburnt coal and decrease the amount of oxygen to penetrate into the char coal and lead to incomplete combustion^38^.

#### EFs from laboratory made semi-coke and bituminous coals

Semi-coke powder is generally an industrial by-product, made from low-rank coal by a low-temperature coal carbonization process (500 °C–700 °C) in the absence of air to distill out synthetic fuels (e.g., unconventional oil and syngas). Comparing quality information of semi-coke briquettes and unprocessed bituminous coals (Tables S3 and S4), it was found that a significant amount of volatile compounds leaves the raw coal due to the carbonization process, i.e., volatile matter content on dry basis was reduced from ~34% in bituminous coal to ~10% in semi-coke. When air pollution control devices such as electrostatic precipitators were installed for industrial carbonization process (TSP emission is regulated by China’s regulation to be lower than 15 mg/m^3^)^39^, PM emission can be significantly reduced. For instance, primary PM_2.5_ removal efficiency in state-owned coking industry is commonly higher than 99%^17^.

For better comparison, we made the semi-coke powder and unprocessed bituminous coal into the same size briquettes (diameter of ~5 cm) with 1.5% adhesion agent and then tested their emissions in Sangpu stove. Table 1 shows their quality information and EFs for these two laboratory made samples. Their corresponding chunks are shown in Fig. S5. After the carbonization process, the volatile matter content was reduced by a factor of ~5. This leads to much lower EFs of primary PM_2.5_, EC, OC, and CO when burning the semi-coke briquette in comparison to burning the unprocessed bituminous coal. SO_2_ and NO_x_ EFs were also affected by the carbonization process, however, the trends are not clear when including data from more samples (as shown in Tables S5 and S6).

Most semi-coke is in powder or in small chunk with a diameter less than 2 cm which is too small for directly residential stove combustion. The briquetting technology is widely applied in China for residential coal application^25^,^26^,^34^. Auxiliary raw materials and/or clays are often added at a certain ratio to make semi-coke briquette for household application. These added materials and clays can affect pollutant EFs. Figure S6 shows primary PM_2.5_ EFs from burning laboratory made semi-coke briquettes without and with 5% additives in Sangpu stove. Four comparative tests all indicate that adding 5% bentonite or MgO in the semi-coke can lower primary PM_2.5_ EF. In this study, we did not explore the influence of manufacturing process on EFs further. Our tested semi-coke briquette samples in next section were not laboratory made and were purchased from several briquette manufacturers. Though different briquette binders and clays might be used by these manufactures, these samples represent the available semi-coke coals on the market.

#### EFs from semi-coke, anthracite, and bituminous coals

**Figure 3:**
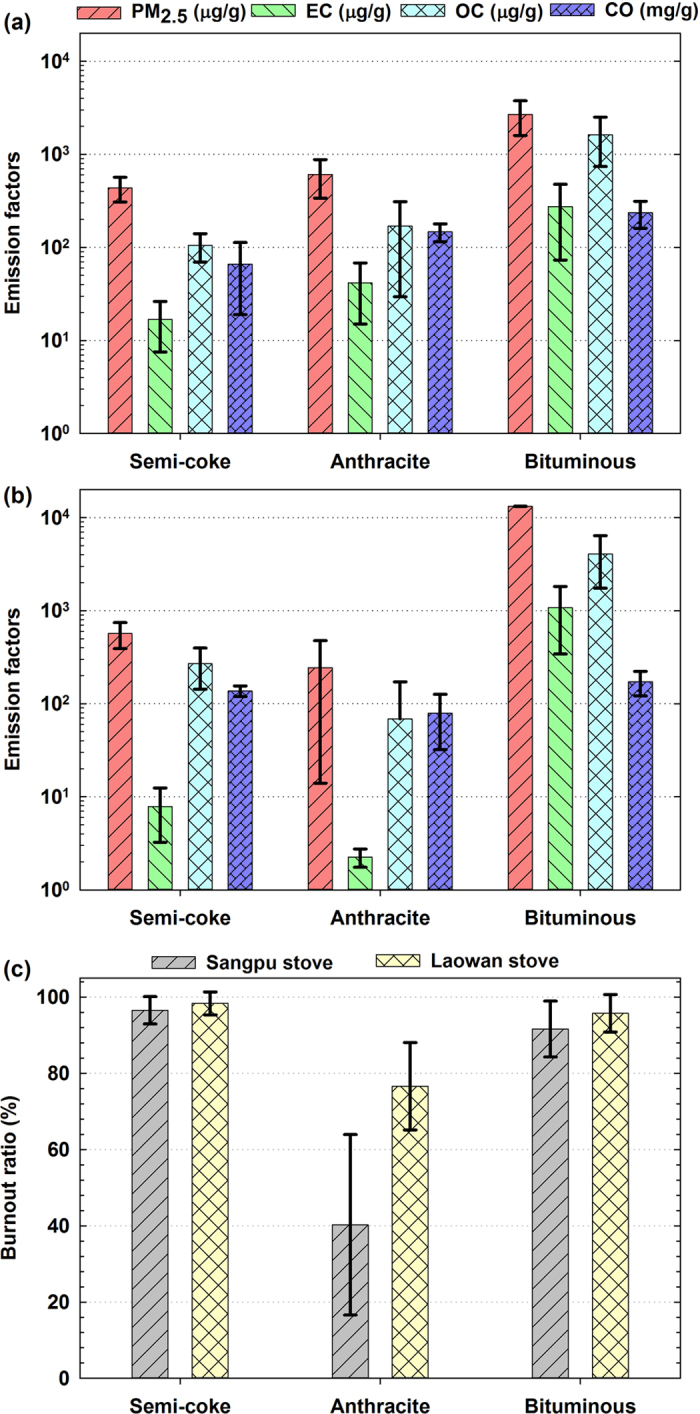
Average emission factors of PM_2.5_, EC, OC, and CO for tested semi-coke, anthracite, and bituminous samples in (a) Laowan stove and (b) Sangpu stove; and (c) average burnout ratios for these three types of coal in the two stoves.

Figure 3 shows the average EFs and burnout ratios for semi-coke, anthracite, and bituminous samples in Laowan and Sangpu stoves (see Tables S3 and S4). Error bars represent one standard deviation in the average EFs for semi-coke, anthracite and bituminous coal samples. As also observed in previous studies^21^,^34^,^40^, emission measurement from residential coal combustion often have high variations which are likely due to many factors, such as coal size, coal forms (chunk or briquette), combustion efficiency, volatile matter content, and coal stacked shape in stove chamber. Although there are variations in burnout ratios and in EFs, these tested semi-coke briquettes in both Laowan and Sangpu stoves perform better than these tested anthracite and bituminous coals. Comparing semi-coke briquettes to anthracite samples, 2-tailed T test analysis shows no significant difference (p > 0.05) for the EFs of primary PM_2.5_, EC, OC, and CO. However, 2-tailed T test analysis shows that semi-coke briquettes have significantly higher burnout ratio than anthracite samples (p = 0.026 and 0.00015 for Sangpu and Laowan stoves, respectively). The low burnout ratio of anthracite in household consumption can lead to the increase in domestic solid waste and redundant operation for stove user. Comparing to bituminous coals, semi-coke briquettes have slightly higher burnout ratios, but much lower EFs of primary PM_2.5_, particulate carbon, and CO.

The variation of primary PM_2.5_, EC, and OC EFs among these three types of coal is mainly due to their volatile contents, as shown in Tables S3 and S4 with referring to Table S1. These comparisons between anthracite and bituminous coals agree with previous reports^23^,^25^,^32^,^33^. Although semi-coke briquette is made from low-rank coal, it has relatively low primary PM_2.5_, EC, and OC EFs because of its low volatile content after carbonization treatment. Its low CO EF is possibly due to its relatively high burning temperature and combustion efficiency. Table S7 shows modified combustion efficiencies (MCE = EF_CO2_/(EF_CO_ + EF_CO2_))^40^,^41^ of those samples tested in Laowan domestic stove. Mean MCEs of 4 tested semi-coke briquettes and 7 tested raw coals are 97.9 ± 0.7% and 94.9 ± 1.6%, respectively.

In addition, semi-coke briquette is easy to be ignited. Taking Laowan stove for example, the times needed for the burning temperature to reach 400 °C are about 20, 20–35, 30–70 minutes for semi-coke, bituminous, and anthracite coals, respectively. The easiness in ignition is governed by coal hardness. Bituminous coal is softer than anthracite coal. Semi-coke made from bituminous coal is further softened due to the increase in porosity during carbonization process^42^.

### Pollution reductions by replacing raw coals with semi-coke briquettes in China

To estimate pollution reduction in China when replacing currently consumed raw bituminous and anthracite coals in household activities with semi-coke briquettes, we made two assumptions. (1) The tested coal samples (semi-coke briquettes, anthracite and bituminous coals) and stoves are representative. (2) According to raw coal production data^23^, bituminous coals and anthracites account for approximately 80% and 20% of China’s residential coal consumption, respectively. With these assumptions and data obtained in our experiments, we performed benefit analysis for the replacement of unprocessed raw coals with semi-coke briquettes in China’s household consumption. Table 2 shows average EFs and the reduction ratios for all three coal types tested in both Laowan and Sangpu stoves. EFs of tested chunks and briquettes of raw coal were estimated. For a given coal type, however, there is no obvious difference in EFs between tested chunk and briquette samples (p > 0.05). Therefore, average EFs including both chunks and briquettes were used to perform reduction estimation. The reduction ratios (R.R.) in EFs by replacing residential raw coals (80% bituminous coals and 20% anthracite coals) with semi-coke briquettes were estimated as follows,


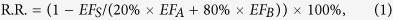


where *EF* stands for a certain emission factor such as primary PM_2.5_, subscripts of *S*, *B*, and *A* stand for semi-coke, bituminous, and anthracite coals, respectively. After the replacement, the reduction ratios in primary PM_2.5_, EC, OC, and CO emissions were estimated to be 92 ± 6%, 98 ± 2%, 91 ± 8%, and 34 ± 21%, respectively. These uncertainties in the emission reduction ratios only reflect error propagation from measured EFs. The uncertainties in assumptions made to estimate emission reduction were not included. They are often difficult to be quantified. In addition, mass-based emission factors were used in the reduction estimation which did not take the differences in fuel energy density and stove thermal efficiency into account. Although gross calorific values for all tested samples (Table S1) have smaller variations comparing to measured emission factors, more laboratory and field measurements are needed to develop energy-based emission factors. 2-tailed T test shows that the replacement leads to significant difference (p < 0.05) for the emissions of PM_2.5_, EC and OC, while the impact on CO emission is not significant (p = 0.25). It indicates that emissions of primary PM_2.5_ and particulate carbon can both be reduced significantly if the replacement is implemented. In addition, the burnout ratio will be improved by ~11% after the replacement, mainly due to the replacement of anthracite coal, which has much lower burnout ratio (the average value for the tested samples and stoves is 66 ± 24%) than bituminous (93 ± 6%) and semi-coke briquettes (97 ± 3%). 2-tailed T test shows that the difference between the burnout ratios of semi-coke briquettes and unprocessed raw coals are significant (p = 0.0076).

## Discussion

If the cost is not considered, switching from household solid fuel to clean forms of energy such as electricity and gas is the best solution for health and environmental benefits^43^. However, taking the economic feasibility of such transition into consideration is also important or even the most important in some cases, especially in developing regions^44^.

In addition to low EFs and high burnout ratio, semi-coke briquettes have lower prices than those of anthracite and bituminous coals. Table 3 shows averaged wholesale prices for ten most requested residential anthracite and bituminous coals. The wholesale price of ten most requested semi-coke powder is ~200 CNY/ton in 2014, relatively lower than chunk coals due to the fact that it is an industrial by-product from bituminous coals. The wholesale price of semi-coke briquettes is ~360 CNY/ton after including cost of adhesion agent and manufacturing. Anthracite coal is often difficult in ignition, has low burnout ratio and a high price. Bituminous coal has lower price than anthracite coal due to their high abundance and production in China. However, the estimated semi-coke briquette price is of some uncertainty considering the influences of different factors such as energy policy, transportation cost, and international energy market.

The semi-coke production was ~28 Mt in 2012 (~19.8 Mt produced in Shaanxi). Shaanxi province planned to increase its production to 30 Mt in 2015 and ~75 Mt in the next few years. It will then meet the total residential coal consumption demand in China. At current production capacity, the government can gradually implement the replacement plan and starts at heavily polluted region such as Beijing-Tianjin-Hebei Region.

Chinese government has strong influence on coal market because state-own companies are key players in both coal production and trade^45^. Thus the replacement of unprocessed raw coals with semi-coke briquettes can happen with few obstacles as long as the government makes the policies. Similar replacement with carbonization processed coals might also be a possible pathway to reduce health risk from household coal consumption and to improve air quality in other coal-producing regions, such as India and South Africa.

By characterizing emissions from burning raw coals and carbonization processed coals in residential heating stoves, this study examined the environmental benefits and feasibility of the proposed replacement of household bituminous and anthracite coals with semi-coke briquettes. In addition to uncertainties discussed above, pollutant emissions from the production of semi-coke are not included in the analysis and need more investigations. Energy-based emission will help to further illustrate the proposed replacement and more studies are needed to develop energy-based emission factors.

## Summary

Household coal combustion is an important anthropogenic emission source of particulate matter and gaseous pollutants in China. Our study of 35 tested coal samples in 2 stoves found that the replacement of unprocessed raw coals by semi-coke briquettes (made from bituminous coals by industrial carbonization treatment with air pollution control devices) for residential coal consumption in China can reduce emission factors of primary PM_2.5_, elemental carbon, and organic carbon significantly. Furthermore, the price of semi-coke is relatively lower than that of unprocessed bituminous and anthracite coals. Additionally, China’s coking industrial capacity can meet the full consumption demand in next few years due to the rapid development of coking industry. These results implicate that the replacement with semi-coke briquettes is a practical pathway to reduce pollution emissions from residential coal combustion and be beneficial for regional and global atmosphere.

We want to end the story with the recommendation from the WHO: “*Unprocessed coal should not be used as a household fuel*”^46^ and our proposal: “*Burning carbonization processed coal is a practical pathway towards reducing pollution emission from household coal combustion*”.

## Methods

### Tested stoves and coal samples

Figure S1 shows photos of the tested stoves (Laowan stove and Sangpu stove), two of the most popular household coal stoves in Beijing (Hereafter “S” denotes that the corresponding content is in the supplementary information.). They are on the recommendation list of the advanced coal stoves by the Beijing government since 2013. Laowan stove is used primarily for heating, while Sangpu stove can be used for both heating and cooking. The advanced stoves can reduce household emissions according to previous investigations^21^,^28^,^29^,^30^,^31^,^47^. With a collaborative program launched in 2012 together with the World Bank, the Chinese government has been actively promoting these advanced household coal stoves. Our study is focused on coal types rather than the stoves.

Quality information of 35 tested residential coal samples is shown in Table S1. Contents of moisture, ash, volatile matter, and fixed carbon were obtained using the standard method of GBT 30732–2014. Total sulfur content was obtained with automatic infrared sulfur analyzer (5E-IRS3000, Changsha Kaiyuan Instruments Co., Ltd). Gross calorific value was obtained using a calorimeter (5E-AC/PL, Changsha Kaiyuan Instruments Co., Ltd). Analysis was conducted at China National Coal Quality Supervision Testing Center and was repeated three times. Table S1 includes unprocessed bituminous and anthracite coals and processed coals (semi-coke). These unprocessed coals were collected in both forms of chunk and briquette (in cake shape) from most popular coal companies, while semi-coke briquette samples were made from semi-coke powders mixing with clay and briquette binders. The semi-coke powders (carbonization processed coal) has been primarily produced in Shaanxi, Inner Mongolia, Shanxi, and Ningxia provinces, while Shaanxi province has produced more than a half of semi-coke powders in China. Semi-coke powders from Shaanxi province were used for making briquettes tested in this study. They were made from bituminous coals from Shaanxi province, and the quality information of the semi-coke samples is close to that of the corresponding bituminous coal sample (e.g., B5ch in Table S1). The tested semi-coke briquettes were prepared by several briquette manufacturers and different briquette binders and clays might be used. In addition, a few laboratory made semi-coke and bituminous briquettes were also listed in Table S1 for controlled EF comparison between processed and unprocessed coals.

### Experimental design and setup

**Figure 4:**
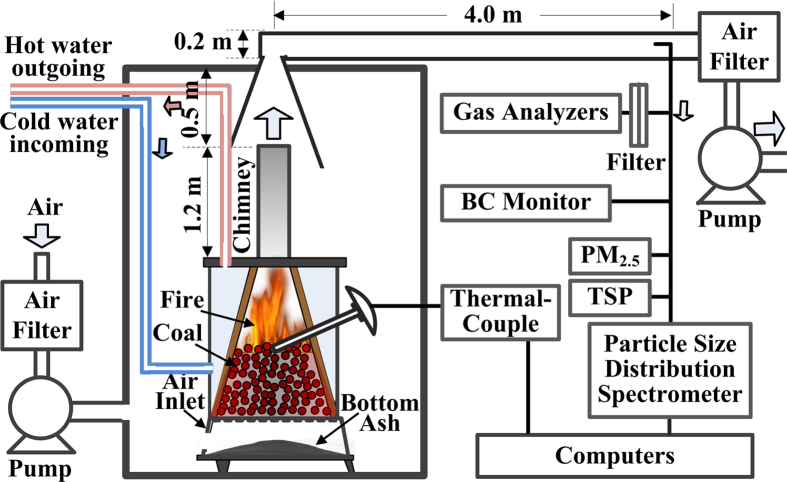
Schematic of the setup for characterizing emissions from household coal combustion. The schematic is drawn using Microsoft Office Visio (Microsoft corp.).

Figure 4 shows the schematic of our setup for characterizing emissions from household coal combustion. Filtered air was pumped into a sealed-room (with a volume of ~ 10 m^3^) containing the test stove, and the room was kept at a slightly positive pressure. An ash door near the stove bottom, used to control the air supply, was fully open to provide air during combustion. An electric thermocouple, whose sensor was put on the burning coal, was used to monitor the burning temperature from fire start to fire extinction. The distance between the chimney exit and the stove chamber is ~1.2 m. The flue gas was collected by a canopy mixing with dilution air from the sealed-room and then drawn into a circular pipe (diameter of 0.22 m) with average flowrate of 9.03 m/s, controlled by a pump.

To represent residential heating process, a water filled radiator (6 kW) was connected to the test stove via a water circulating pump. The high power phase (the first two hours after fire start) often heats water to more than 80 °C. The radiator’s highest temperature was controlled to be less than 80 °C by releasing hot water and introducing cold water. The coal weight was fixed at 10.0 kg and 3.0 kg for Laowan and Sangpu stoves, respectively. Coal samples were ignited by propane gas with a fixed flow rate 3 L/min. Each tested sample was lighted until the stove chamber to be about 400 °C. The ignition consumes propane gas of ~0.1–0.5 m^3^. Emission sampling lasted from fire start to fire extinction (referring to coal burning temperature). Comparing to the emission from coal combustion, the contribution of the propane gas is negligible. Comparing to other solid fuel combustion^30^, a complete coal burning needs a longer time due to the slow burning rate, especially in the fire extinction phase (see Fig. 2). Each coal burning cycle ranges from 6 to 10 hours and from 5 to 8 hours for Laowan and Sangpu stoves, respectively.

EFs of total suspended particles (TSP), primary PM_2.5_, EC, and CO were measured. Three or two successful tests of full burning cycles were conducted for each sample in each stove. Aerosol instruments include a PM_2.5_ cyclone (16.7 L/min, URG-2000-30 EH), a TSP sampler (10.0 L/min), and a home-made particle size distribution spectrometer for the size range of 3 nm–10 μm^48^, and a BC monitor (micro-Aeth, Model AE51, Magee Scientific, CA). Quartz filters were used to collect primary PM_2.5_ and TSP samples. Organic carbon and elemental carbon in PM_2.5_ samples were measured using a thermal-optical method in an OC/EC analyzer (DRI 2001A). CO, SO_2_, NO_x_ concentrations in the flue gas were monitored using gas analyzers (Thermo Scientific™ model 48i, 42i, and 43i). Aerosol mass concentration of the filtered ambient air pumped into the sealed-room was less than 1 μg/m^3^, much lower than the averaged aerosol concentration during coal combustion (on the order of 100 μg/m^3^). Thus the influence of ambient air on PM_2.5_ and TSP EFs is negligible. Ambient CO concentration was subtracted during CO EF calculation

Heating and cooking are two common household coal consumption activities in China. To represent the cooking practice, top cover of Sangpu stove was removed and fire flame then went out of its top. EFs for cooking and heating practices are comparatively shown in Table S2. The cooking practice generally emits more PM_2.5_ than the heating practice. Since the cooking practice is relatively non-stable and more complex^30^, our analysis focused on emissions from burning different types of coal for the heating practice.

### EF calculation

Primary PM_2.5_ EF was estimated as follows,


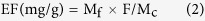


where M_f_ (mg) and M_c_ (g) are PM_2.5_ mass collected on the quartz filter and burned coal weight, respectively; F is the ratio of sampling flow rate to total flow rate in the tunnel.

EC and OC EFs were estimated as follows,


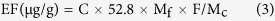


where C (μg/cm^2^) is the mean EC or OC concentration in tested PM_2.5_ quartz filter with the sampling area of 52.8 cm^2^.

EFs of CO, SO_2_, and NO_2_ were estimated as follows,


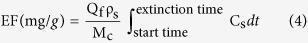


where C_s_ (ppm) is measured concentration of CO, SO_2_, or NO_2_; ρ_s_ is the species density; Q_f_ is the gas flowrate in the tunnel.

### Burnout ratio

During household combustion, coal might not be burnt out completely into ashes, especially for anthracite coal. Burnout ratio is used to represent the completeness of the coal combustion during a burning cycle. Coal burnout ratio, η_br_, was estimated as,


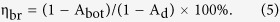


where A_bot_ is the ratio of bottom ash weight after household coal combustion to the coal sample weight; and A_d_ is the ratio of bottom ash weight after complete coal combustion to the coal sample weight^49^, which are obtained during coal proximate analysis and reported as coal quality information in Table S1. Burnout ratios for tested semi-coke, anthracite, and bituminous samples in two stoves are presented in Tables S3 and S4.

## Additional Information

**How to cite this article**: Li, Q. *et al.* Semi-coke briquettes: towards reducing emissions of primary PM_2.5_, particulate carbon, and carbon monoxide from household coal combustion in China. *Sci. Rep.* **6**, 19306; doi: 10.1038/srep19306 (2016).

## Supplementary Material

Supplementary Information

## Figures and Tables

**Figure 1:**
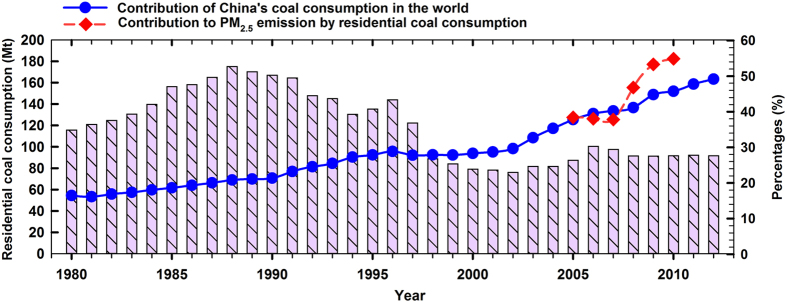
China’s residential coal consumption, the percentage of primary PM_2.5_ emission from residential coal consumption in that from all coal consumption activities in China, and the percentage of China’s coal consumption in global total coal consumption. *Source*: World Coal Association^16^, China Energy Statistical Yearbook^50^, and PM_2.5_ emission data from BAU model^17^.

**Table 1:** Quality information and EFs of bituminous briquette (B8br in Table S1) and its corresponding semi-coke briquette (S3br).

Coal quality information & EFs	Bituminous (Unprocessed coal)	Semi-coke (Processed coal)
M_*d*_ (%)^a^	0.84	2.28
A_*d*_ (%)^a^	8.86	12.13
V_*daf*_ (%)^a^	35.72	6.91
S_*t,d*_ (%)^a^	0.35	0.37
Q_*gr, ad*_ (kJ/g)^a^	29.25	29.32
FC_*ad*_ (%)^a^	58.08	81.81
PM_2.5_ (mg/g)	25.49 ± 2.30	1.06 ± 0.07
EC (μg/g)	1352 ± 712	9 ± 1
OC (μg/g)	5885 ± 1604	402 ± 142
CO (mg/g)	208 ± 5	93 ± 12
SO_2_ (mg/g)	0.36 ± 0.08	0.44 ± 0.04
NO_2_ (mg/g)^b^	0.97 ± 0.03	0.49 ± 0.03

^*a*^obtained by proximate analysis (see Table S1 for details).

^*b*^For mass emission factor, NO_x_ was calculated as NO_2_.

**Table 2:** Averaged emission factors for three types of coal in two stoves and the reduction ratios (R.R.) after the replacement of all unprocessed anthracite and bituminous coals with semi-coke briquettes.

Coal	Typle	PM_2.5_(mg/g)	EC (μg/g)	OC (μg/g)	CO (mg/g)
Raw anthracite	Chunk	0.24 ± 0.09	16 ± 13	40 ± 49	95 ± 41
	Briquette	0.53 ± 0.24	27 ± 24	161 ± 92	96 ± 35
	Average	0.40 ± 0.23	22 ± 20	109 ± 97	96 ± 35
Raw bituminous	Chunk	6.54 ± 6.30	678 ± 760	2485 ± 2309	189 ± 72
	Briquette	13.23 ± 3.16	647 ± 9	4025 ± 363	210 ± 4
	Average	7.87 ± 6.32	672 ± 671	2793 ± 2141	193 ± 63
Semi-coke	Briquette	0.49 ± 0.18	12 ± 8	194 ± 127	115 ± 45
	R.R. (%)	92 ± 6	98 ± 2	91 ± 8	34 ± 31

**Table 3:** Averaged wholesale coal prices (CNY/ton) for unprocessed raw coals and processed semi-coke briquettes.

Coal	Semi-coke briquette	Anthracite	Bituminous
Price	360 ± 120	700 ± 240	550 ± 220

*Source:* Alibaba.com (the largest online trading platform in China, accessed 24^th^ November, 2014).
