# Supplementary material for: Semi-coke briquettes: towards reducing emissions of primary PM2.5, particulate carbon, and carbon monoxide from household coal combustion in China
Source: Sci Rep. 2016 Jan 19;6:19306. doi: 10.1038/srep19306 (PMC4726058; doi:10.1038/srep19306)
Supplement: Supplementary Information [file srep19306-s1.pdf]

Supplementary Information

Semi-coke briquettes: towards reducing emissions of primary $PM_{2.5}$, particulate carbon, and carbon monoxide from household coal combustion in China

Qing Li [1], Xinghua Li [2], Jingkun Jiang [1,3*], Lei Duan [1], Su Ge [4], Qi Zhang [1], Jianguo Deng [1], Shuxiao Wang [1] and Jiming Hao [1,3*]

[1] State Key Joint Laboratory of Environment Simulation and Pollution Control, School of Environment, Tsinghua University, Beijing, 100084, China.

[2] School of Chemistry and Environment, Beihang University, Beijing 100191, China.

[3] State Environmental Protection Key Laboratory of Sources and Control of Air Pollution Complex, Beijing 100084, China.

[4] College of Environmental Science and Engineering, Nankai University, Tianjin, 300071, China.

*To whom correspondence should be addressed:

J. Jiang and J. Hao, School of Environment, Tsinghua University, Beijing, 100084, China.

Email: jiangjk@tsinghua.edu.cn (JJ) and hjm-den@tsinghua.edu.cn (JH).

7 tables

6 figures

14 pages

Table S1. Coal quality information for all tested samples in this study. The coal quality information was obtained by proximate analysis. All the samples are listed in the order of increasing V_{daf} which stands for the content of volatile matter on dry and ash-free basis.

Parameter[b] / Sample[a]	M_d (%)	A_d (%)	V_d (%)	V_{daf} (%)	$S_{t,d}$ (%)	$Q_{gr,ad}$ (kJ/g)
S1br	1.92	11.61	3.51	3.97	0.33	29.50
S2br	1.57	15.06	3.47	4.09	0.24	27.87
S3br	2.28	12.13	6.06	6.91	0.37	29.32
S4br	1.32	16.23	7.64	9.12	0.41	28.31
S5br	1.55	15.66	7.73	9.17	0.61	28.27
S6br	1.64	23.76	7.06	9.25	0.42	23.35
S7br	1.77	16.81	8.2	9.85	0.44	25.95
S8br	2.03	17.82	8.35	10.20	0.34	26.87
S9br	5.04	18.75	7.78	10.20	0.30	24.42
S10br	5.01	16.91	8.76	10.50	0.24	27.05
S11br	2.10	15.52	9.13	10.81	0.44	25.32
S12br	1.37	16.04	9.97	11.87	0.73	28.34
S13br	1.73	21.93	9.71	12.40	0.37	24.62
S14br	6.00	9.87	10.60	12.60	0.33	27.14
S15br	2.48	15.77	13.68	16.24	0.42	24.71
A1ch	6.20	13.93	2.27	2.84	0.86	27.39
A2ch	2.15	7.16	3.64	4.01	0.28	32.49
A3br	1.18	10.61	5.22	5.92	0.3	31.29
A4br	1.43	19.7	5.14	6.52	0.3	24.92
A5ch	2.08	19.46	5.52	7.04	0.22	24.99
A6br	2.08	28.37	5.24	7.31	0.35	23.05
A7br	1.35	12.85	6.67	7.38	0.24	30.17
A8ch	1.09	22.28	5.78	7.54	0.29	26.14
A9br	1.68	24.38	5.58	7.55	0.32	24.5
A10br	1.11	12.06	6.93	7.88	0.32	31.21
A11ch	2.10	20.52	7.02	9.07	0.24	26.28
A12br	2.94	22.83	9.75	12.63	0.35	24.83
B1ch	7.20	7.09	25.30	29.50	0.35	26.41
B2br	0.30	9.68	30.42	33.68	0.33	29.14
B3ch	8.50	3.70	29.95	34.11	0.10	28.52
B4ch	6.31	6.80	29.80	34.30	0.16	27.86
B5ch	3.34	4.10	33.40	34.80	0.22	28.1
B6ch	1.40	19.85	28.50	35.50	0.44	26.82
B7ch	0.81	9.76	32.90	35.50	0.36	28.98
B8br	0.84	8.86	33.06	35.70	0.35	29.25

[a] S, A, and B in the sample indexes stand for semi-coke, anthracite, and bituminous coals, respectively, while br and ch stand for briquette and chunk, respectively. Main materials (semi-coke powders) for making 15 semi-coke samples were made from coals in northern Shaanxi province.

[b] M_d, A_d, V_d, $S_{t,d}$, and $Q_{gr,ad}$ stand for moisture, ash, volatile matter, total sulfur, and gross calorific value on dry basis.

Table S2. Comparison of emission factors during heating and cooking modes in Sangpu domestic stove (ZS60A, see Figure S1(b)).

Sample	Burning mode[a]	A_{bot} (%)	η_{br} (%)	$PM_{2.5}$ (mg/g)	CO (mg/g)
S7br	Heating	0.12	100	0.60	113
	Cooking	0.12	100	0.85	107
A9br	Heating	0.41	76.3	0.59	142
	Cooking	0.40	77.7	0.95	129
A10ch	Heating	0.77	20.1	0.10	48
	Cooking	0.77	20.1	0.26	43

[a] Experiments were repeated for 2-3 times for each sample. The values shown here are the average from each experiment.

Table S3. Emission factors and burnout ratio for the tested samples in Laowan stove (NS18C, see Figure S1(a)). See Table S1 for abbreviations and definitions.

Sample[a]	A_{bot}[a] (%)	η_{br}[b] (%)	$PM_{2.5}$ (mg/g)	BC (μg/g)	OC (μg/g)	CO (mg/g)
S8br	18.00	99.76	0.60	25	92	86
S9br	19.50	98.98	0.35	9	72	156
S10br	16.56	100.0	0.23	3	74	51
S13br	26.70	93.02	0.46	25	156	33
S14br	9.96	99.89	0.35	19	118	NA[c]
A2ch	36.70	66.88	0.23	35	17	95
A3ch	30.50	76.37	0.35	22	27	148
A4br	54.00	54.36	0.59	46	274	94
A6br	38.95	84.06	0.23	11	61	NA
A7ch	36.10	71.11	0.32	17	139	NA
A8br	41.00	73.98	0.48	49	108	117
A9br	40.00	77.70	0.73	64	178	84
A10ch	31.00	76.62	0.25	16	22	NA
A11br	26.00	92.44	0.64	30	172	100
A12br	28.00	92.33	0.81	10	254	NA
B1ch	6.00	100.0	1.16	67	581	117
B4ch	14.00	88.65	3.40	156	2295	283
B5ch	4.85	98.80	1.68	449	792	227
B6ch	22.27	95.32	3.62	546	2584	NA
B7ch	15.00	90.86	2.64	94	1308	255

[a] A_{bot} = the ratio of the mass of collected bottom ash to the coal mass, where the bottom ash was collected after the coal combustion extinction;

[b]burnout ratio: $\eta_{br} = (1- A_{bot})/(1 - A_d) \times 100\%$.

[c] NA = measurement was not conducted or not successful.

Table S4. Emission factors and burnout ratio for the tested samples in Sangpu domestic stove (ZS60A, see Figure S1(b)). See Table S1 and S3 for abbreviations and definitions.

Sample	A_{bot} (%)	η_{br} (%)	$PM_{2.5}$ (mg/g)	BC (μg/g)	OC (μg/g)	CO (mg/g)
S1br	0.14	100	0.41	10	260	133
S2br	0.23	91.5	0.53	10	216	132
S3br	0.19	95.1	0.60	4	137	110
S4br	0.25	98.7	0.62	4	288	163
S8br	0.21	93.7	0.87	15	507	138
S9br	0.18	100	0.38	4	214	148
A1ch	0.59	46.6	0.16	2	19	89
A5br	0.81	17.9	0.13	2	15	38
A9br	0.41	76.2	0.59	3	223	142
A10ch	0.77	20.1	0.10	2	17	48
B2br	0.11	97.3	10.99	653	3768	207
B3ch	0.15	83.1	7.93	379	941	100
B4ch	0.15	87.7	13.33	1667	3889	135
B5ch	0.10	97.0	18.53	2064	7489	204
B7br	0.11	98.3	15.46	640	4282	213

Table S5. Emission factors of SO_2 and NOx for the tested samples in Laowan domestic stove (NS18C, see Figure S1(a)). NO_x was converted into NO_2. See Table S1 for sample names and their information.

Sample	SO_2 (mg/g)	NO_2 (mg/g)
S8br	4.41	0.43
S9br	2.09	0.66
S10br	NA	NA
S13br	NA	NA
S14br	NA	NA
A2ch	3.42	0.61
A3ch	4.41	1.67
A4br	0.48	0.28
A6br	NA	NA
A7ch	NA	NA
A8br	4.32	0.95
A9br	3.04	0.61
A10ch	NA	NA
A11br	2.17	0.46
A12br	NA	NA
B1ch	2.33	0.89
B4ch	5.24	1.49
B5ch	NA	NA
B6ch	NA	NA
B7ch	5.52	0.60

Table S6. Emission factors of SO_2 and NOx for the tested samples in Sangpu domestic stove (ZS60A, see Figure S1(b)). NO_x was converted into NO_2. See Table S1 for sample names and their information.

Sample	SO_2 (mg/g)	NO_2 (mg/g)
S2br	0.48	0.86
S4br	0.62	0.80
S5br	1.89	0.75
S6br	0.94	0.31
S13br	6.00	0.80
S14br	0.50	0.98
A1ch	6.99	0.25
A5br	0.03	0.12
A9br	3.95	1.10
A10ch	2.55	1.10
B2br	0.72	1.00
B3ch	0.87	0.11
B4ch	1.13	0.49
B5ch	0.64	1.00
B7br	0.28	0.95

Table S7. EFs for CO_2 and CO and modified combustion efficiency (MCE = $\Delta[CO_2]/(\Delta[CO_2]+ \Delta[CO])$, where $\Delta[CO_2]$ and $\Delta[CO]$ are the fire-integrated excess molar mixing ratios of CO_2 and CO) for the tested samples in Laowan domestic stove (NS18C, see Figure S1(a)).

Sample	CO (mg/g)	CO_2 (g/g)	MCE (%)
S8br	86	6.98	98.1%
S9br	156	7.66	96.9%
S10br	51	4.14	98.1%
S13br	33	3.19	98.4%
A2ch	95	3.68	96.1%
A3ch	148	3.09	93.0%
A4br	94	1.85	92.6%
A9br	84	3.44	96.3%
A11br	100	3.87	96.1%
B1ch	117	4.53	96.1%
B4ch	283	7.50	94.4%

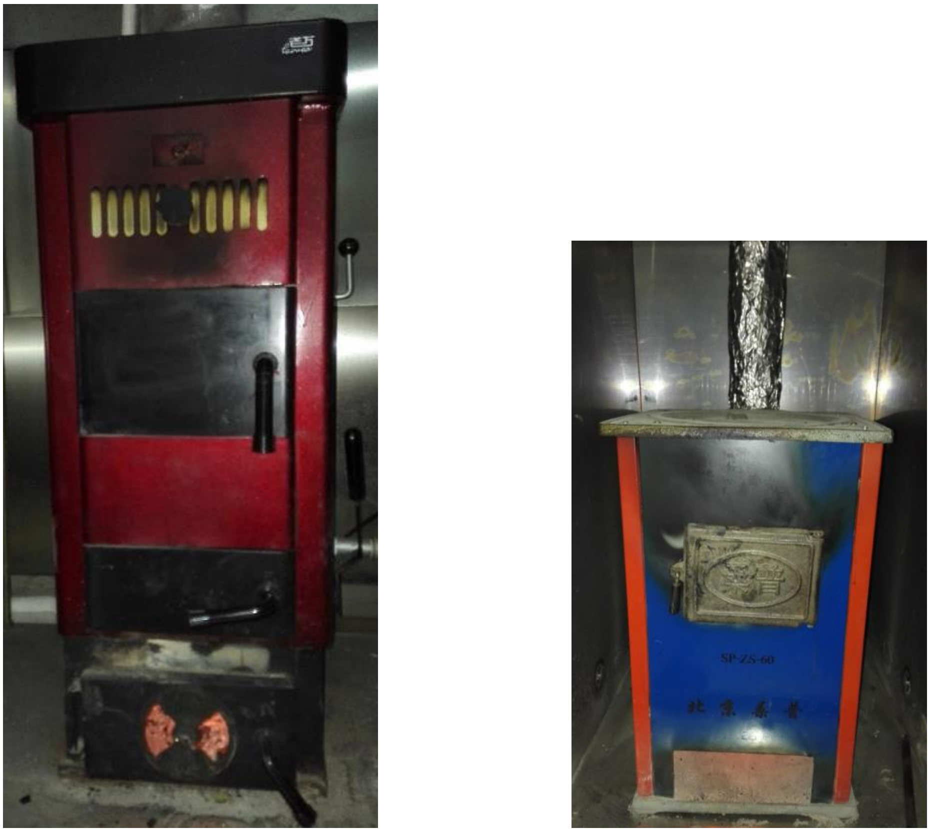

(a) Laowan NS18C **(b)** Sangpu ZS60A

Fig. S1 Photos of two tested domestic stoves (a) Laowan (NS18C) and (b) Sangpu (ZS60A). Laowan stove is only used for heating, while Sangpu stove can be used for both heating and cooking. Rated output powers are (a) 18 kW and (b) 4.7 kW, respectively. Recommended heating areas are (a) 150 m^2 and (b) 60 m^2. Stove thermal efficiencies are (a) 70% and (b) 65%. These two photos were taken by the authors using Canon digital camera during this study.

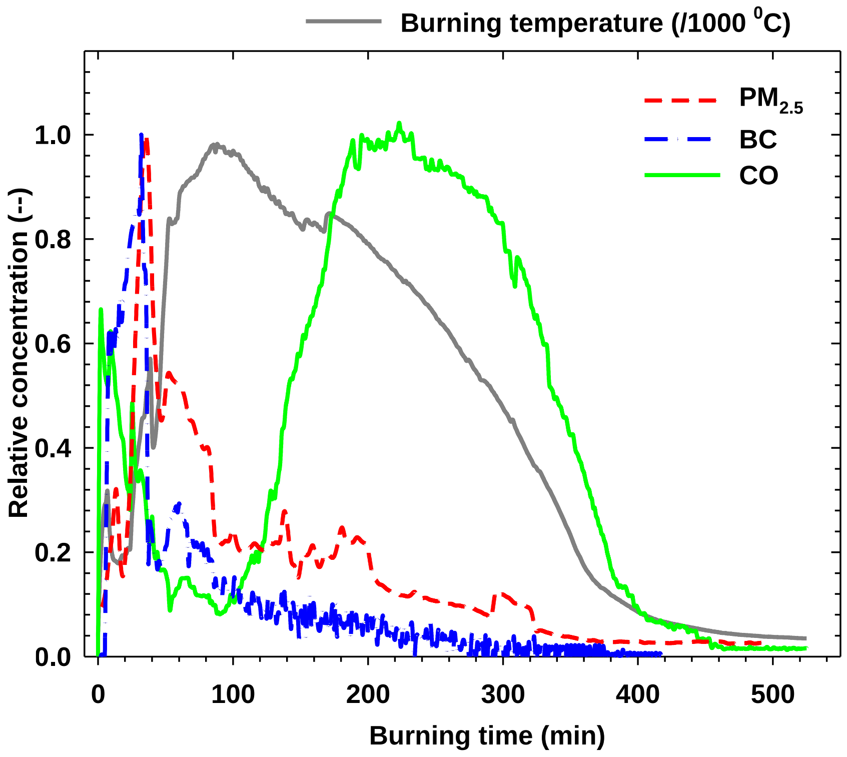

Fig. S2 Typical emission profiles of primary $PM_{2.5}$, BC, and CO with reference to the burning temperature from fire start to fire extinction (anthracite sample A2ch in Laowan stove).

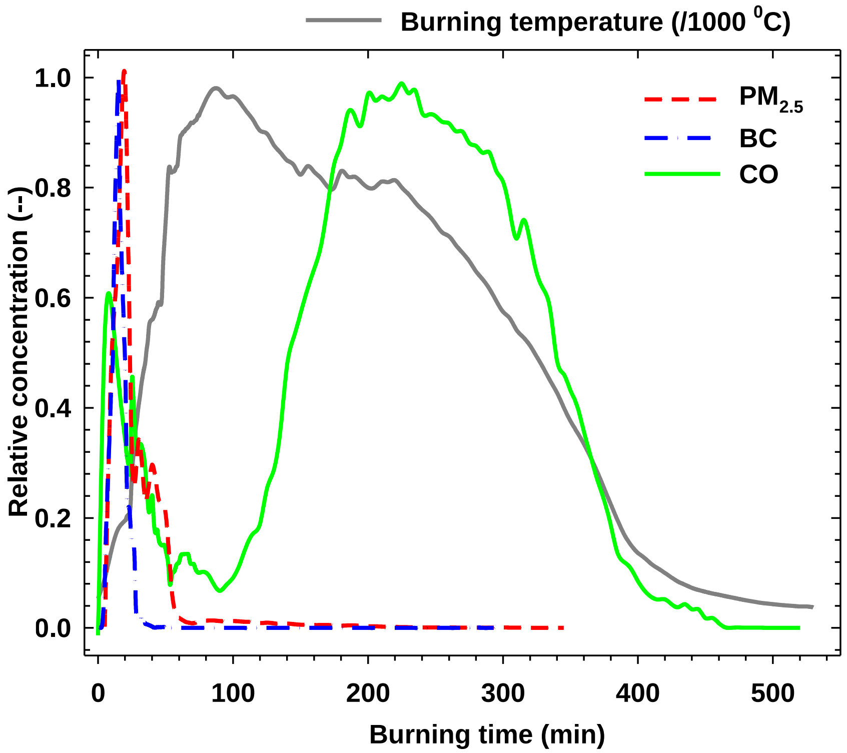

Fig. S3 Typical emission profiles of primary $PM_{2.5}$, BC, and CO with reference to the burning temperature from fire start to fire extinction (bituminous sample B5ch in Laowan stove).

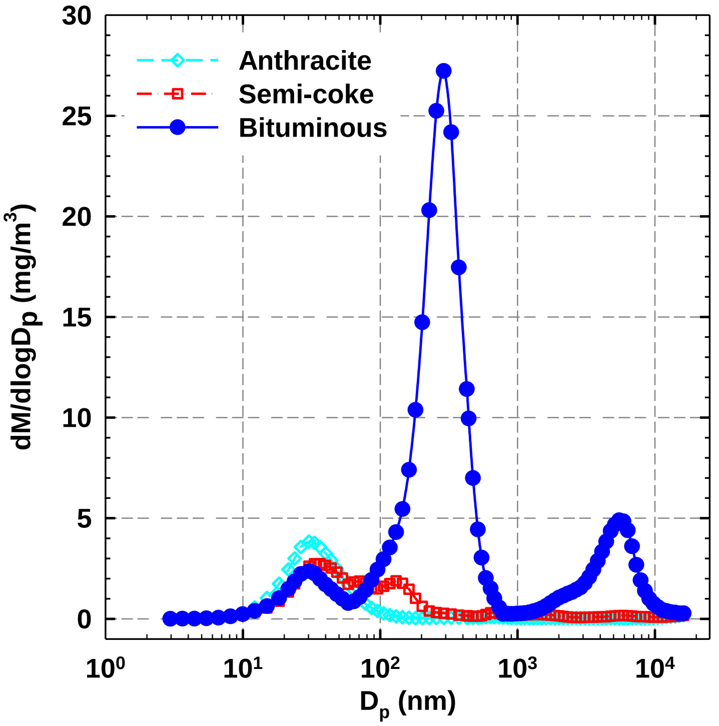

Fig. S4 Three typical aerosol mass distributions when burning bituminous (sample B5ch), anthracite (sample A2ch), and semi-coke (sample S9br) in Laowan stove. They were measured by the particle size distribution spectrometer.

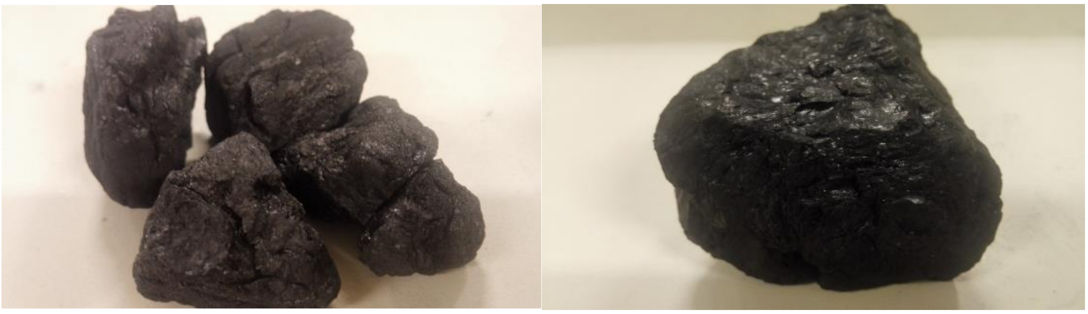

Fig. S5 Photos of semi-coke chunk (left) and its raw coal chunk (right). Comparing to raw coal chunk, semi-coke chunk has higher porosity resulting from the escape of volatile compounds during low temperature carbonization process. These two photos were taken by Canon digital camera during this study.

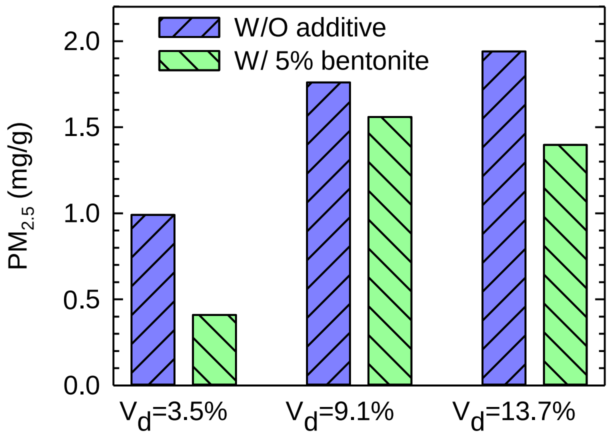

Fig. S6 Primary $PM_{2.5}$ emission factors of semi-coke briquettes without (W/O) and with (W/) 5% bentonite (tested in Sangpu stove). Variations in volatile matter (V_d) of these laboratory made semi-coke briquettes are mainly due to different amounts of adhesion agent were used. These EFs from three tested sample groups (i.e., S1br, S11br, and S15br) suggest that additives can reduce primary $PM_{2.5}$ emission.
